# Super-Resolution Localisation of Nuclear PI(4)P and Identification of Its Interacting Proteome

**DOI:** 10.3390/cells9051191

**Published:** 2020-05-11

**Authors:** Veronika Fáberová, Ilona Kalasová, Alžběta Krausová, Pavel Hozák

**Affiliations:** 1 Department of Biology of the Cell Nucleus, Institute of Molecular Genetics of the Czech Academy of Sciences, Vídeňská 1083, 142 20 Prague, Czech Republic; faberova@img.cas.cz (V.F.); ilona.kalasova@img.cas.cz (I.K.); alzbeta.krausova@img.cas.cz (A.K.); 2 Department of Epigenetics of the Cell Nucleus, Institute of Molecular Genetics of the Czech Academy of Sciences, division BIOCEV, Průmyslová 595, 252 20 Vestec, Czech Republic; 3 Microscopy Centre of the Institute of Molecular Genetics of the Czech Academy of Sciences, Vídeňská 1083, 142 20 Prague, Czech Republic

**Keywords:** nucleus, phosphoinositides, PI(4)P

## Abstract

Phosphoinositides are glycerol-based phospholipids, and they play essential roles in cellular signalling, membrane and cytoskeletal dynamics, cell movement, and the modulation of ion channels and transporters. Phosphoinositides are also associated with fundamental nuclear processes through their nuclear protein-binding partners, even though membranes do not exist inside of the nucleus. Phosphatidylinositol 4-phosphate (PI(4)P) is one of the most abundant cellular phosphoinositides; however, its functions in the nucleus are still poorly understood. In this study, we describe PI(4)P localisation in the cell nucleus by super-resolution light and electron microscopy, and employ immunoprecipitation with a specific anti-PI(4)P antibody and subsequent mass spectrometry analysis to determine PI(4)P’s interaction partners. We show that PI(4)P is present at the nuclear envelope, in nuclear lamina, in nuclear speckles and in nucleoli and also forms multiple small foci in the nucleoplasm. Nuclear PI(4)P undergoes re-localisation to the cytoplasm during cell division; it does not localise to chromosomes, nucleolar organising regions or mitotic interchromatin granules. When PI(4)P and PI(4,5)P2 are compared, they have different nuclear localisations during interphase and mitosis, pointing to their functional differences in the cell nucleus. Mass spectrometry identified hundreds of proteins, including 12 potentially novel PI(4)P interactors, most of them functioning in vital nuclear processes such as pre-mRNA splicing, transcription or nuclear transport, thus extending the current knowledge of PI(4)P’s interaction partners. Based on these data, we propose that PI(4)P also plays a role in essential nuclear processes as a part of protein–lipid complexes. Altogether, these observations provide a novel insight into the role of PI(4)P in nuclear functions and provide a direction for further investigation.

## 1. Introduction

Phosphoinositides (PIs) are glycerol-based phospholipids with a negative charge. They are amphipathic molecules consisting of hydrophilic inositol heads and hydrophobic fatty acyl tails. Seven different PIs can be produced by the phosphorylation and dephosphorylation of the inositol heads at the 3′, 4′ and 5′ positions. PIs are important molecules; they are involved in cell and membrane dynamics, vesicular transport and cell signalling [1,2,3,4]. Close to 15% of phosphoinositides also localise to the nucleus. Some PIs are associated with the nuclear membrane; however, a great portion of PIs localise to distinct regions of the nuclei [5] such as the interchromatin and chromatin regions, and nucleoli [6,7,8,9,10,11,12,13]. Phosphoinositides play a role in various nuclear functions, which is in agreement with their diverse nuclear localisation. PIs are implicated in DNA transcription, pre-rRNA and pre-mRNA processing, cell differentiation, the DNA damage response and apoptosis (reviewed in [14,15]). Others and we have previously shown that PIs interact with both RNA Pol I and II and regulate the recruitment and activity of transcription factors, as well as gene expression at the epigenetic level [13,16,17,18,19,20,21,22,23,24]. The majority of these studies concentrate on the functions of PI(4,5)P2, despite the fact that phosphatidylinositol 4-phosphate (PI(4)P) and PI(4,5)P2 are the most abundant PIs [1]; PI(4)P localisation and its role in the nucleus remain largely unknown.

In the cytoplasm, PI(4)P localises to the Golgi apparatus, endosomes and plasma membrane. It is involved in the regulation of intracellular trafficking between these organelles [1,25,26]. Previously, we detected nuclear PI(4)P with a specific anti-PI(4)P antibody [27], and in this study, we investigated the localisation and possible functions of nuclear PI(4)P in greater detail. We showed that PI(4)P is present at the nuclear membrane, in nuclear speckles, in nucleoli and in small nucleoplasmic foci. We then analysed PI(4)P lipid–protein complexes by immunoprecipitation with an anti-PI(4)P antibody followed by a proteomic analysis. Around 100 nuclear proteins were identified as participating in important nuclear processes such as pre-mRNA splicing, transcription or nuclear transport, indicating an essential, yet undiscovered, role for PI(4)P in the biology of the cell nucleus.

## 2. Materials and Methods

### 2.1. Cell Culture

U2OS cells were cultured in DMEM medium supplemented with 10% FBS, under 5% CO_2_, at 37 °C, in a humidified atmosphere. HeLa suspension cells were cultured in RPMI medium supplemented with 5% FBS, under 5% CO_2_, at 37 °C, in a humidified atmosphere on a spinner.

### 2.2. Antibodies

The primary antibodies used in this study are as follows: anti-PI(4)P (Echelon Biosciences Inc., Salt Lake City, UT, USA, Z-P004, 10 μg/mL for immunofluorescence (IF), 2.5 μg for immunoprecipitation (IP)), anti-Son (Abcam, Cambridge, UK, ab121759, 1 μg/mL for IF), anti-C23 (Abcam, ab22758, 1 μg/mL for IF), anti-lamin B1 (Abcam, ab16048, 3 μg/mL for IF), anti-PI(4,5)P2 (Echelon Biosciences Inc., Z-A045, 2.5 μg/mL for IF), anti-RPA194 (Santa Cruz Biotechnology, Inc., Dallas, TX, USA, sc-28714, 1 μg/mL for IF), anti-mouse IgM isotype control (Abcam, ab91545, 2.5 μg for IP), anti-hnRNP U (Merck, Darmstadt, Germany, 05-1516, 1 μg/mL for Western blot (WB)), anti-NXF1 (Abcam, ab129160, 0.03 μg/mL for WB) and anti-NUMA1 (Abcam, ab109262, 0.1 μg/mL for WB).

The secondary antibodies used in this study are as follows: goat anti-Mouse IgM Alexa Fluor 555 (Invitrogen, Waltham, MA, USA, A21426, 5 μg/mL), goat anti-Mouse IgM Alexa Fluor 568 (Invitrogen, A21043, 5 μg/mL) and goat anti-Rabbit IgG Alexa Fluor 488 (Invitrogen, A11034, 5 μg/mL) for IF; and IRDye^®^ 800 CW Donkey anti-Rabbit IgG (LI-COR Biosciences, Lincoln, NE, USA, 926-32213, 0.05 μg/mL) and IRDye^®^ 800 CW Donkey anti-Mouse IgG (LI-COR Biosciences, 926-32212, 0.05 μg/mL) for WB.

### 2.3. Recombinant Protein Purification

The OSH1-PH domain was expressed and purified as described previously using a pET-42a GST-OSH1-PH-His construct [27].

### 2.4. Pull-Down, Immunoprecipitation and Western Blots

Nuclear extract was prepared, as described previously [28], from HeLa suspension cells. For pull-down, PI-coated agarose beads were washed three times in lysis buffer (50 mM Tris, pH = 7.5; 150 mM NaCl; 1% NP-40; cOmplete) and blocked with 5% BSA in lysis buffer. The BSA was aspirated, and the beads were incubated with 1 mg of the protein from the nuclear extract for 2 h at 4 °C on a roller shaker. The beads were then washed three times with lysis buffer, and the proteins were eluted in 2× Laemmli buffer. For immunoprecipitation, Dynabeads™ MyOne™ Tosylactivated beads coupled with IgM isotype control, anti-PI(4)P or anti-PI(4,5)P2 antibodies were incubated with 1 mg of the protein from the nuclear extract for 4 h at 4 °C on a roller shaker. The beads were then washed three times with lysis buffer, and the proteins were eluted in 2× Laemmli buffer or the beads were dried and used for proteomic analysis.

Proteins were loaded on a 10% polyacrylamide gel, separated by SDS-PAGE and transferred onto a nitrocellulose membrane. The membranes were blocked with 3% BSA in PBS and then incubated with primary antibodies and with appropriate secondary antibodies conjugated to IRDye. The signal was detected with the Odyssey Infrared Imaging System (Li-COR Biosciences).

### 2.5. Proteomic Analysis

#### 2.5.1. Protein Digestion

IP samples were resuspended in 100 mM TEAB containing 2% SDC. Cysteines were reduced with a 5 mM final concentration of TCEP (60 °C for 60 min) and blocked with a 10 mM final concentration of MMTS (10 min RT). The samples were cleaved on beads with 1 µg of trypsin at 37 °C overnight. After digestion, the samples were centrifuged and the supernatants were collected and acidified with TFA to a 1% final concentration. The SDC was removed by ethylacetate extraction [29]. The peptides were desalted using in-house made stage tips packed with C18 disks (Empore™ 3M, Saint Paul, MN, USA) according to Rappsilber et al. [30].

#### 2.5.2. Nano-Scale Chromatographic Tandem Mass Spectrometry (nLC-MS 2) Analysis

A nano-reversed phase column (EASY-Spray™ column, 50 cm × 75 µm ID, PepMap C18, 2 µm particles, 100 Å pore size, Thermo Fisher Scientific, Waltham, MA, USA) was used for LC/MS analysis. The mobile phase buffer A was composed of water and 0.1% formic acid. The mobile phase B was composed of acetonitrile and 0.1% formic acid. The samples were loaded onto the trap column (Acclaim PepMap300, C18, 5 µm, 300 Å Wide Pore, 300 µm × 5 mm, 5 Cartridges) for 4 min at 15 μL/min. The loading buffer was composed of water, 2% acetonitrile and 0.1% trifluoroacetic acid. The peptides were eluted with a mobile phase B gradient from 4% to 35% B over 60 min. Eluting peptide cations were converted to gas-phase ions by electrospray ionisation and analysed on a Thermo Orbitrap Fusion (Q-OT- qIT, Thermo Fisher Scientific). Survey scans of peptide precursors from 350 to 1400 *m*/*z* were performed at a 120K resolution (at 200 *m*/*z*) with a 5 × 10^5^ ion count target. Tandem MS was performed by isolation at 1,5 Th with the quadrupole; HCD fragmentation, with a normalised collision energy of 30; and rapid scan MS analysis, in the ion trap. The MS/MS ion count target was set to 10^4^, and the maximum injection time was 35 ms. Only those precursors with a charge state of 2–6 were sampled for MS/MS. The dynamic exclusion duration was set to 45 s with a 10 ppm tolerance around the selected precursor and its isotopes. Monoisotopic precursor selection was turned on. The instrument was run in top speed mode with 2 s cycles [31].

#### 2.5.3. Data Analysis

All data were analysed and quantified with the MaxQuant software version 1.6.2.1 [32]. The false discovery rate (FDR) was set to 1% for both proteins and peptides, and we specified a minimum length of seven amino acids. The Andromeda search engine was used for the MS/MS spectra search against the Human database (downloaded from Uniprot on September 2017, containing 20,142 entries). The enzyme specificity was set as C-terminal to Arg and Lys, also allowing cleavage at proline bonds and a maximum of two missed cleavages. The dithiomethylation of cysteine was selected as a fixed modification, and N-terminal protein acetylation and methionine oxidation, as variable modifications. The “match between runs” feature of MaxQuant was used to transfer identifications to other LC-MS/MS runs based on their masses and retention times (maximum deviation, 0.7 min), and this was also used in quantification experiments. Quantifications were performed with the label-free algorithms described recently. Data analysis was performed using the Perseus 1.6.1.3 software.

### 2.6. Indirect Immunofluorescence Microscopy

Cells grown on glass coverslips were washed with PBS three times, then fixed and permeabilised simultaneously with 0.1% Triton X-100 and 4% formaldehyde in PBS for 10 min at room temperature (RT). The samples were blocked with 5% normal goat serum in PBS for 1 h at RT, incubated with primary antibodies diluted in PBS for 1 h at RT and then washed with PBS. The samples were then incubated with appropriate secondary antibodies diluted in PBS for 1 h at RT, washed with PBS and mounted in 90% glycerol and 1% 1,4-diazabicyclo-octane (DABCO).

Images were acquired using a super-resolution Leica TCS SP8 STED 3X microscope with a 100× (NA = 1.4) oil immersion objective and the LAS X software version 3.5.5. The acquired images were deconvolved using the Huygens Professional software.

**Figure 1:**
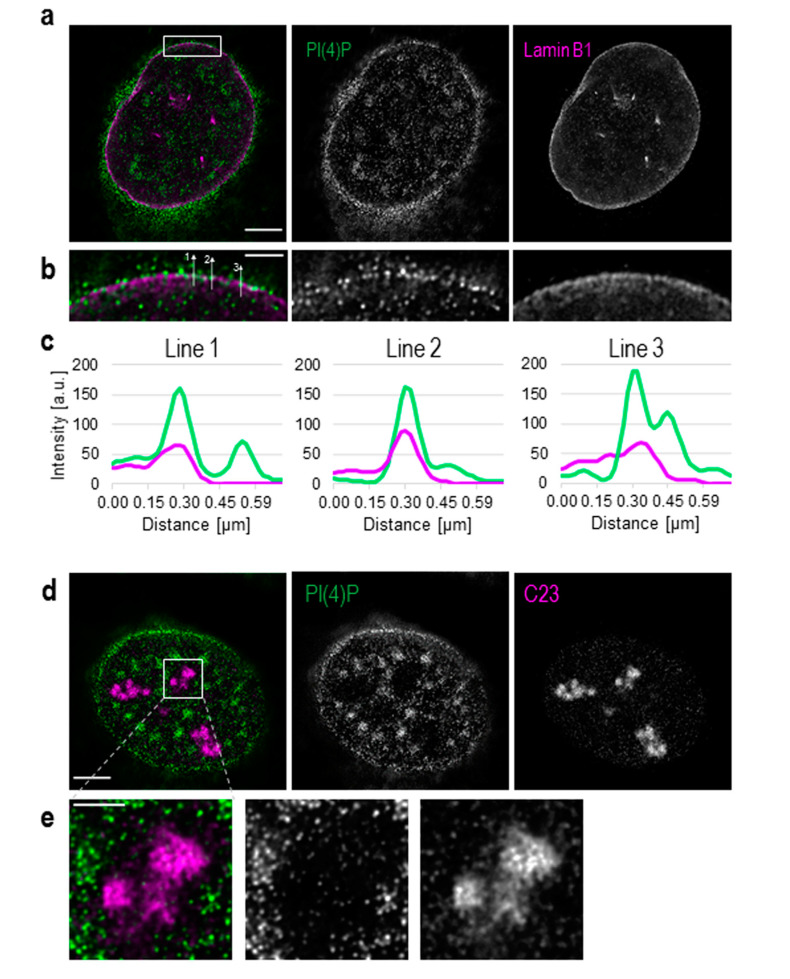
Detection of phosphatidylinositol 4-phosphate (PI(4)P) at the nuclear envelope and in the nucleoli. Super-resolution STED microscopy revealed the localisation of PI(4)P in the nuclear lamina, the nucleoli, nuclear speckles and nucleoplasmic foci. (**a**) Fluorescently labelled PI(4)P and lamin B1. (**b**) PI(4)P co-localises with lamin B1. (**c**) Intensities of pixels along the lines in **b**, PI(4)P (green) and lamin B1 (purple). Lines are drawn in a direction from the nucleoplasm to the cytoplasm (from bottom to top). Graphs were made using the FiJi software. (**d**) Localisation of PI(4)P and a protein C23, a marker of the nucleoli. (**e**) PI(4)P localises to the nucleoli, but the intensity of PI(4)P staining is much lower than in the nucleoplasm. Scale bars: a—5 μm; b—1 μm; d—5 μm; e—2 μm.

The graphs in Figure 1c were made using the FiJi software with a custom-made macro using *Analyze › Plot Profile*, which shows pixel intensities along a line selection in RGB images.

The quantification of the portion of the PI(4)P signal in nuclear speckles was done using the FiJi software. We created masks based on the DAPI (nucleus) and Son signals (nuclear speckles). We masked the PI(4)P channel with the DAPI mask to get the overall PI(4)P signal in the cell nucleus, then we masked this picture with the Son mask to get the portion of PI(4)P signal in the nuclear speckles. We used the masked pictures to calculate Raw Integrated Density using *Analyze › Set Measurements › Integrated density*. Fifteen cells were analysed; each cell was composed of five z-stacks. The portion of the nuclear speckle PI(4)P signal is presented as a ratio: the PI(4)P signal in the nuclear speckles/the PI(4)P signal in the nucleus.

### 2.7. Electron Microscopy

HeLa cells were fixed in 3% formaldehyde with 0.1% glutaraldehyde, dehydrated in an ethanol series and embedded in LR White resin. Ultrathin sections prepared using a Leica UC6 ultramicrotome and Diatome 45° diamond knife were immunolabelled and contrasted according to Stradalova et al. [33] using mouse anti-PI(4)P antibody (Echelon Biosciences, Z-P004, 20 μg/mL) and goat anti-mouse IgM conjugated to 6 nm gold nanoparticles (Jackson ImmunoResearch, 115-195-075, dilution 1:30). For double staining (Figure 4), ultrathin sections were incubated with purified OSH1-PH domain tagged with GST, washed and then immunolabelled using anti-PI(4,5)P2 antibody (Echelon Biosciences, Z-A045, 16 μg/mL), rabbit polyclonal anti-GST antibody (a gift from Igor Shevelev, 5 μg/mL) and the secondary antibodies goat anti-rabbit IgG conjugated to 6 nm gold nanoparticles (Jackson ImmunoResearch, 111-195-144, dilution 1:30) and goat anti-mouse IgM conjugated to 12 nm gold nanoparticles (Jackson ImmunoResearch, 115-205-075, dilution 1:30).

Images were acquired using a Jeol JEM 1400 Flash, operated at 120 KV, equipped with a Hamamatsu Orca Flash CMOS camera.

Clustering and co-localisation analyses were performed using our self-developed Gold plugin [34] for the Ellipse software version 2.0.8.1 (ViDiTo, Slovakia).

## 3. Results

### 3.1. PI(4)P Localises to the Nuclear Envelope, Nucleoli, Nuclear Speckles and Nucleoplasmic Foci

**Figure 2:**
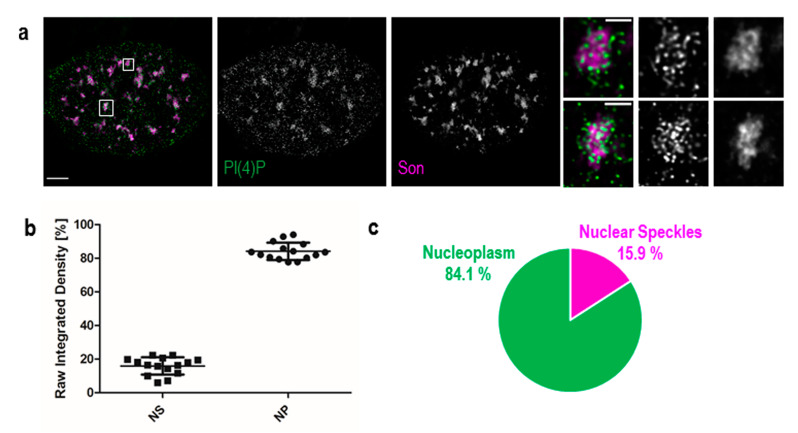
PI(4)P also localises to the nuclear speckles. Beside the nuclear membrane, the nuclear lamina, the nucleoli and nucleoplasmic foci, super-resolution STED microscopy revealed the localisation of PI(4)P in the nuclear speckles. (**a**) Co-localisation of PI(4)P with the Son protein, a marker of the nuclear speckles. PI(4)P is enriched inside and at the edges of nuclear speckles. Scale bar: 5 μm, insets 1 μm. (**b**) Percentage of PI(4)P signal in the nuclear speckles and the nucleoplasm per nucleus. Nearly 16% of the nuclear PI(4)P signal comes from the nuclear speckles. The graph was made in the GraphPad Prism program; data are shown as the mean with SD. NS—nuclear speckles, NP—nucleoplasm. (**c**) Pie chart representation of the results in **b**.

**Figure 3:**
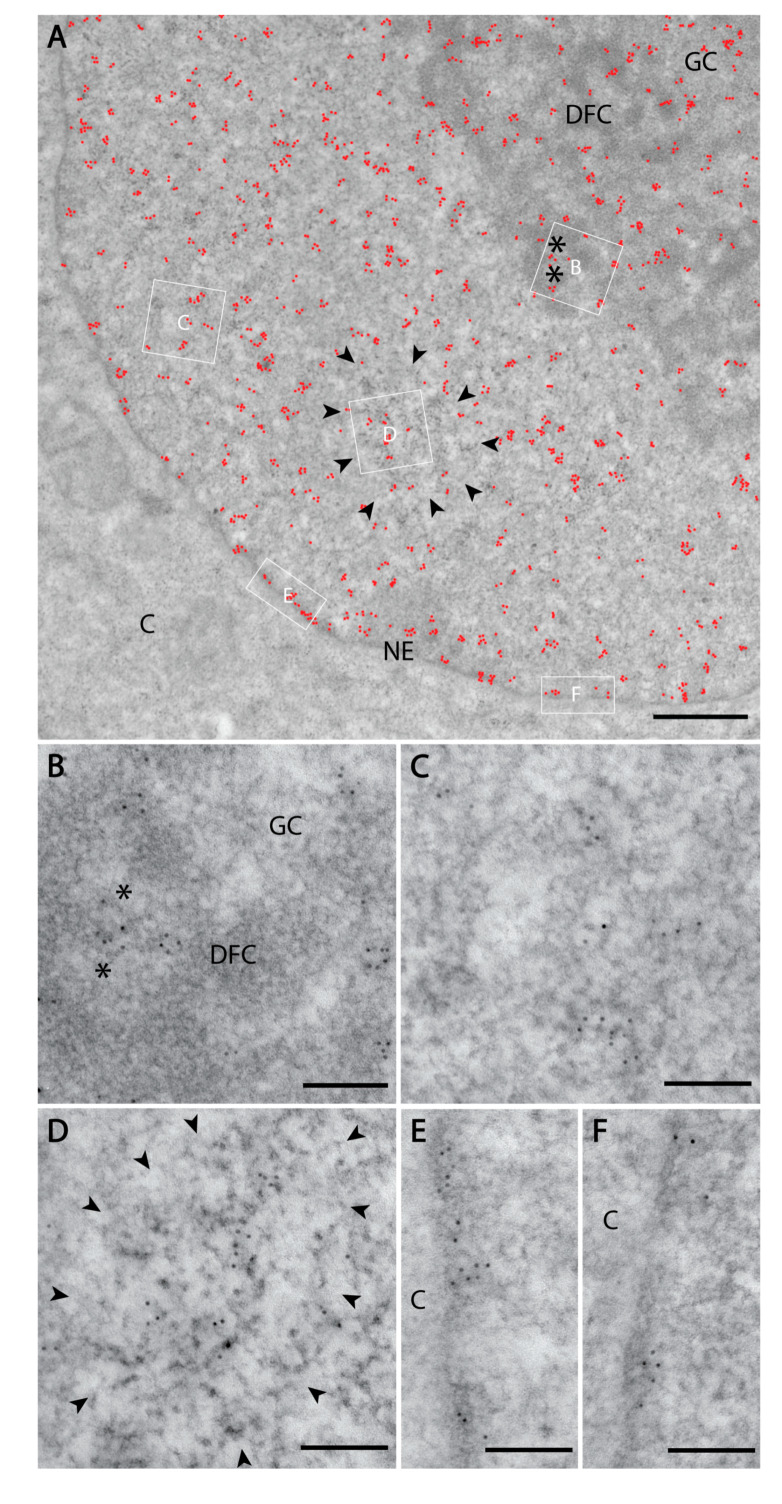
Nuclear PI(4)P localisation according to transmission electron microscopy. Electron microscopy revealed the localisation of PI(4)P: (**A**) to the cell nucleus (red dots). Black asterisk—fibrillar centre; DFC—dense fibrillar component; GC—granular component; NE—nuclear envelope; C—cytoplasm; black arrowheads delineate nuclear speckles. (**B**) In the nucleolus, PI(4)P is enriched in the DFC and also GC, but it does not localise to the FC. (**C**) In the nucleoplasm, PI(4)P is enriched on chromatin. (**D**) PI(4)P is also enriched at the nuclear speckles, and nuclear envelope (**E**,**F**) where it localises to nuclear lamina. (**B**–**F**) are high magnification insets from the same cell.

**Figure 4:**
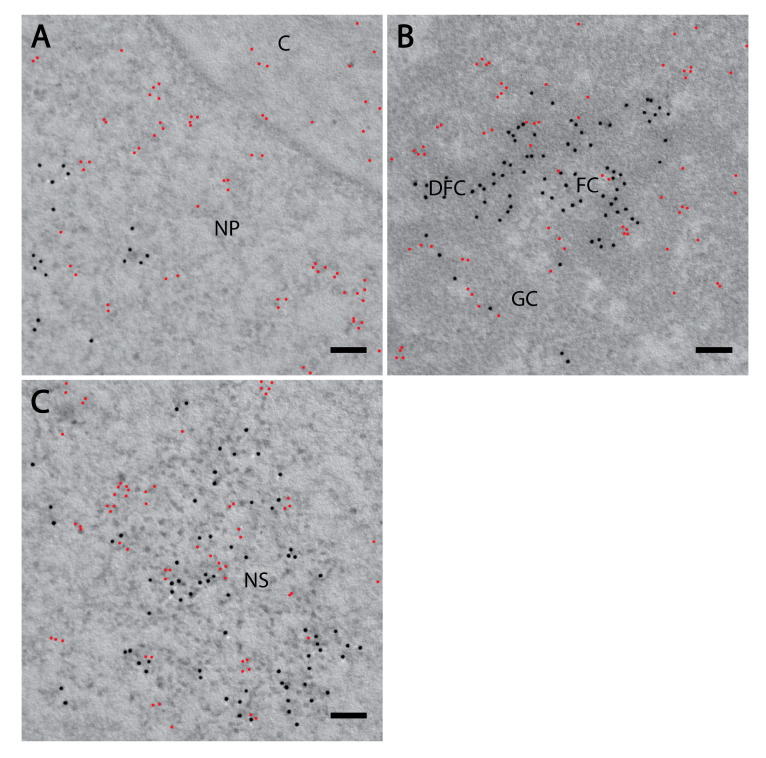
PI(4)P forms different foci than PI(4,5)P2. Electron microscopy showed the localisation of PI(4)P (red) and PI(4,5)P2 (black) are different in the nucleoplasm. Localisation of PI(4)P visualised with OSH1-PH domain and PI(4,5)P2 visualised with antibody (**A**) in the nucleoplasm and nuclear envelope, (**B**) in the nucleolus and (**C**) in the nuclear speckles. NP—nucleoplasm, C—cytoplasm, FC—fibrillar centre, DFC—dense fibrillar component, GC—granular component, NS—nuclear speckle. Scale bars: 500 nm.

**Figure 5:**
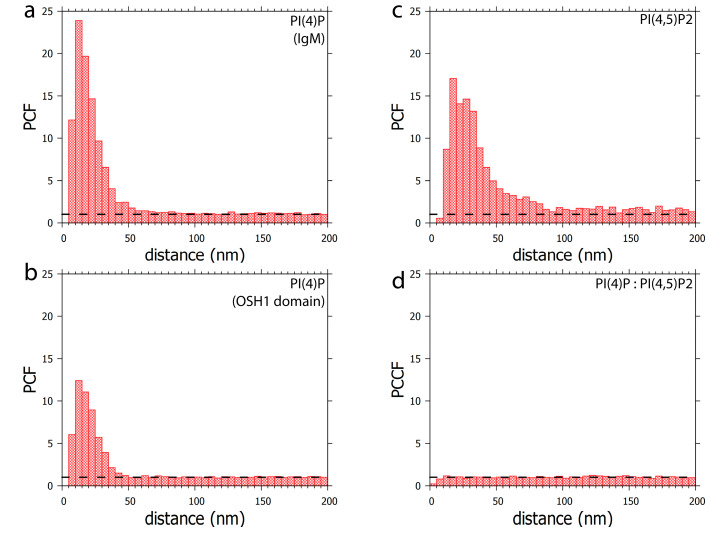
Clustering patterns of (**a**) PI(4)P labelled with anti-PI(4)P antibody or (**b**) PI(4)P labelled with OSH1-PH domain. The foci formed by PI(4)P are up to 50 nm in size. (**c**) Clustering pattern of PI(4,5)P2 labelled with anti-PI(4,5)P2 antibody. The foci formed by PI(4,5)P2 are up to 100 nm in size (**d**) Co-localisation analysis of PI(4,5)P2 with OSH1-PH labelling (Figure 4). PI(4)P does not specifically co-localise with PI(4,5)P2. PCF—pair correlation function, PCCF—pair cross-correlation function. Dashed lines mark the value of functions equal to 1, which corresponds to a random distribution.

We have previously tested the specificity of the anti-PI(4)P antibody, and we detected PI(4)P in the nuclei of U2OS cells [27]. Here, we investigated PI(4)P localisation in greater detail using super-resolution light (Figure 1 and Figure 2) and electron microscopy (Figure 3). We also compared the localisation of PI(4)P with that of PI(4,5)P2, whose nuclear localisation has been already described (Figure 4 and Figure 5; [18,19]). Because of the same host and isotype of the anti-PI(4)P and anti-PI(4,5)P2 antibodies, we used the anti-PI(4,5)P2 antibody to visualise PI(4,5)P2, and the OSH1-PH domain to visulalise PI(4)P for the co-localisation experiments for these two phosphoinositides (Figure 4 and Figure 5 [27]).

Besides its well-described cytoplasmic localisation, PI(4)P also localises to the cell nucleus (Figure S1). We show that PI(4)P is present at the nuclear membrane where it co-localises with nuclear lamina, as detected with the anti-lamin B1 antibody (Figure 1a–c). A portion of PI(4)P also localises to the nucleoli, as detected with the anti-C23 antibody (Figure 1d,e).

Additionally, we used Son, a splicing cofactor and a core component of nuclear speckles [35,36], to investigate the PI(4)P signal in these structures (Figure 2a). The distribution of PI(4)P in nuclear speckles is not uniform; it mostly forms foci inside of nuclear speckles and seems to avoid dense regions marked by Son (Figure 2a insets). Besides, the positive foci of PI(4)P are often present at the edges of nuclear speckles (Figure 2a insets), where the active transcription of some genes takes place [37,38,39]. It can be seen that PI(4)P is mostly enriched in nuclear speckles; therefore, we decided to quantify the portion of the overall nuclear PI(4)P signal in nuclear speckles. We show that 15.9% of PI(4)P resides in nuclear speckles whereas the remaining 84.1% of PI(4)P localises outside of nuclear speckles, in nucleoplasm and nucleolus (Figure 2b,c).

We further employed transmission electron microscopy to study PI(4)P in greater detail. We confirmed the localisation of PI(4)P to the nuclear speckles (Figure 3a,d) and at the nuclear lamina (Figure 3a,e,f). Moreover, P(4)P forms foci within the nucleolus (Figure 3a). The foci are predominantly enriched in the dense fibrillar component (DFC) and partially in the granular component (GC) too (Figure 3b). On the other hand, the majority of PI(4,5)P2 localises to the fibrillar centre (FC) and DFC regions (Figure 4b, [18]). These observations might point to different nucleolar functions of PI(4)P and PI(4,5)P2. Eventually, PI(4,5)P2 might be cleaved to PI(4)P during rDNA transcription, rRNA processing and ribosome assembly.

Furthermore, PI(4)P forms discrete foci dispersed throughout the nucleoplasm (Figure 1, Figure 2 and Figure 3 and Figure 4a). We performed a cluster analysis, which revealed that the foci formed by PI(4)P in the nucleoplasm are up to 50 nm in size (Figure 5a,b). On the other hand, the foci formed by PI(4,5)P2 are up to 100 nm in size (Figure 5c, [19]). PI(4)P forms smaller foci compared to PI(4,5)P2; however, these foci do not differ only in size; the morphology and the localisation of the foci are different, too. The PI(4)P foci are not as homogenous as the PI(4,5)P2 foci and they are enriched on chromatin (Figure 3c). We also observed the distinct localisation of PI(4,5)P2 and PI(4)P in the nucleoplasm (Figure 4a). The PI(4)P foci and the PI(4,5)P2 foci do not co-localise in the nucleoplasm (Figure 5d).

In conclusion, we identified the specific nuclear localisation of PI(4)P. We show that PI(4)P localises to the nuclear lamina and the nuclear speckles and forms nucleolar foci localised mostly in DFC but also in the GC. The edge between the fibrillar centres (FC) and DFC is known as a place for active rDNA transcription [40], indicating a possible role for PI(4)P in this process. Furthermore, PI(4)P forms 50 nm large foci, which are enriched on chromatin in the nucleoplasm and typically do not co-localise with PI(4,5)P2. These observations indicate the possible implications of PI(4)P in essential nuclear processes, such as nuclear transport, rDNA transcription, and rRNA and mRNA processing.

### 3.2. Localisation of PI(4)P through the Cell Cycle

**Figure 6:**
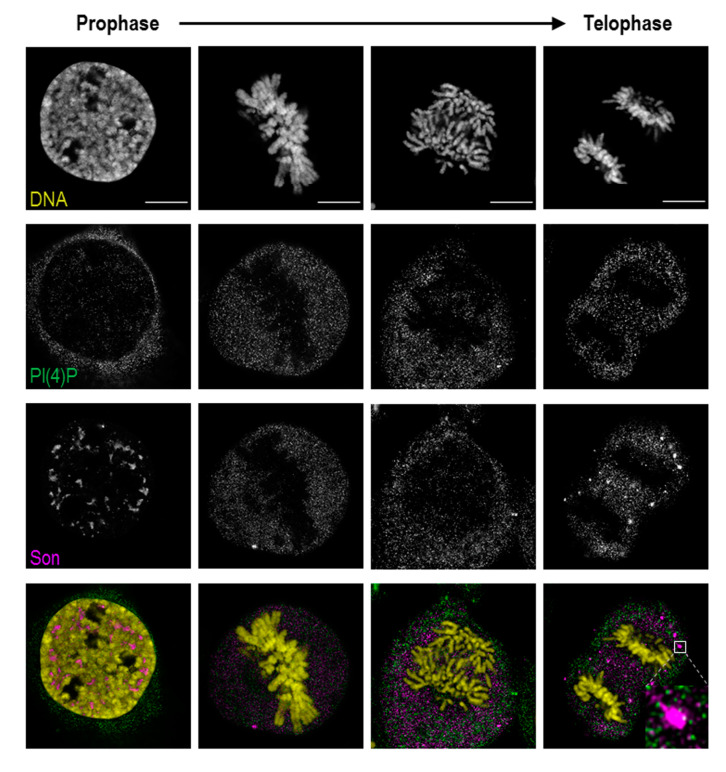
PI(4)P is dispersed in the cytoplasm during mitosis. PI(4)P and Son were labelled, and their localisation was followed across mitotic stages by super-resolution STED microscopy. PI(4)P does not localise to chromatin, nucleolar organising regions (NORs) or mitotic interchromatin granules (MIGs) during cell division. DNA was stained by DAPI. Scale bars: 10 μm.

During mitosis, nuclear speckle-associated proteins generally become diffusely distributed throughout the cytoplasm. As mitosis progresses, the nuclear speckle-associated proteins accumulate in mitotic interchromatin granules (MIGs); [37,41]. PI(4,5)P2 localises to these structures as well as to nucleolar organising regions (NORs); [9,18]. The distinct localisation of PI(4)P and PI(4,5)P2 within the nuclear compartments in the interphase described here prompted us to determine the localisation of PI(4)P during mitosis using light super-resolution microscopy (Figure 6).

In prophase, PI(4)P still localises to the nuclear speckles, but the signal from nucleoplasmic foci is decreased. In later stages of mitosis, when PI(4,5)P2 localises to NORs and MIGs, PI(4)P is diffused throughout the whole cytoplasm (Figure S2). PI(4)P does not co-localise with either RNA Pol I, a marker of NORs, or Son, specifically localising to MIGs (Figure 6, inset; Figure S2).

Altogether, PI(4)P, in contrast to PI(4,5)P2, undergoes different nuclear re-localisations during mitosis. The localisation of PI(4)P within the MIGs and the NORs is lost during mitosis, while PI(4)P resides in the cytoplasm. These observations indicate that the PI(4)P localisation is strictly regulated during mitosis, and we again observed distinct PI(4)P and PI(4,5)P2 behaviours, further supporting the differences in function between these two phosphoinositides.

### 3.3. PI(4)P is in Complex with a Number of Nuclear Proteins

**Figure 7:**
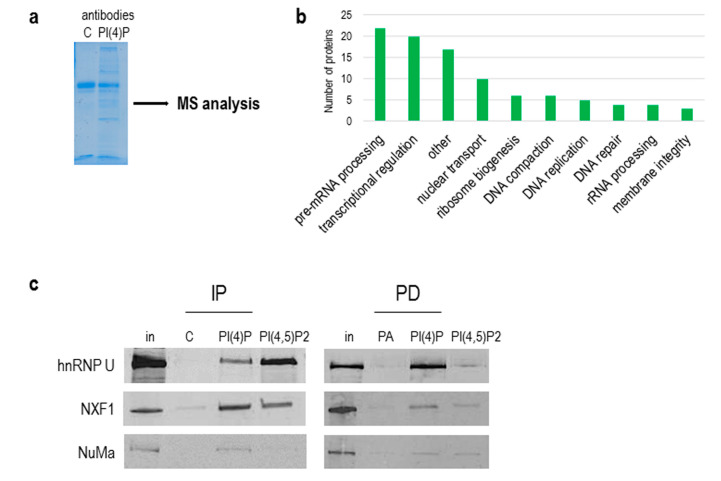
PI(4)P forms lipid–protein complexes in the nucleus. (**a**) PI(4)P-binding partners were immunoprecipitated with anti-PI(4)P antibody, and the gel was stained with Coomassie Blue; MS—mass spectrometry. (**b**) The graph shows a number of proteins identified by mass spectrometry, divided into different sub-groups based on their function in the nucleus (uniprot.org). (**c**) Example of proteins immunoprecipitated with anti-PI(4)P and anti-PI(4,5)P2 antibody (IP) and pulled down with PI(4)P/PI(4,5)P2-coated agarose beads (PD). Ten percent of an input nuclear lysate was loaded on the gel in rows “in”. In—input; C—control mouse IgM antibody; PA—phosphatidic acid-coated agarose beads; hnRNP U—heterogeneous nuclear ribonucleoprotein U; NXF1—nuclear RNA export factor 1; NuMa—nuclear mitotic apparatus protein 1.

As we showed above, the natures of PI(4,5)P2 and PI(4)P differ, and since PI(4,5)P2 plays an important role in RNA Pol I and II transcription [13,19], we continued with the identification of PI(4)P-interacting partners in the cell nucleus. To do so, we used a nuclear extract from asynchronous HeLa suspension cells, immunoprecipitated proteins with antibodies, and performed mass spectrometry (MS) analysis of immunoprecipitated complexes (Figure 7a). We used control IgM, anti-PI(4)P and anti-PI(4,5)P2 antibodies for the experiment. We selected and compared only proteins that were enriched in PI(4)P and PI(4,5)P2 fractions compared to the control IgM fraction (Table S1). We identified 97 nuclear proteins. The majority of the identified proteins are involved in pre-mRNA processing, predominantly splicing. The other identified proteins are involved in transcriptional regulation, nuclear transport and ribosome biogenesis (Figure 7b). Among the 97 proteins, there were 23 proteins enriched more in the PI(4)P fraction than in the PI(4,5)P2 fraction (highlighted in orange in Table S1), and 12 proteins were found only in the PI(4)P fraction (highlighted in blue in Table S1). For example, replication protein A 14 kDA subunit (RPA3) is a part of a heterotrimeric replication protein A complex that is required for DNA replication and repair [42].

Besides an immunoprecipitation with anti-PI(4)P and anti-PI(4,5)P2 antibodies, we used pull-down assays with PI(4)P and PI(4,5)P2-coupled beads to verify the proteomics data. We were able to specifically immunoprecipitate and pull-down proteins hnRNP U, NXF1 and NuMa and detect them by Western blots (Figure 7c).

Collectively, we compared the protein interactomes of PI(4)P and PI(4,5)P2 in the nuclear extract of the HeLa cell line, revealing almost 100 nuclear proteins identified as possible novel interacting partners. Importantly, proteins associated with PI(4)P are components of the RNA transcription and processing machineries, as well as those of DNA replication, indicating the likely role of PI(4)P in these processes.

## 4. Discussion

The presence of the phosphoinositides (PIs) in the cell nucleus was reported over 25 years ago [43,44]. Until now, scientists have observed multiple roles for PIs in nuclear processes, such as DNA transcription, pre-mRNA processing and DNA damage (reviewed in Castano et al. [14]). However, the lack of knowledge of the nuclear metabolism, localisation throughout the cell cycle and interacting partners of the PIs is still a challenge in determining the functions of these components.

Here, we show the nuclear localisation of PI(4)P during the cell cycle at the super-resolution level. PI(4)P localises specifically to a few nuclear compartments—at the nuclear lamina, in the nucleolus, in the nuclear speckles and in small nucleoplasmic foci (Figure 1, Figure 2 and Figure 3). It was reported that phosphoinositides make up only 4% of the lipid content of rat liver nuclei. The major nuclear lipids are phosphatidylcholine (PC) and phosphatidylethanolamine (PE); [45,46]. We showed that PI(4)P is a part of the nuclear lamina (Figure 1a–c and Figure 3e–f). We detected PI(4)P within the dense fibrillar component (DFC) and granular component (GC) of the nucleoli (Figure 1d,e and Figure 3a,b). The nucleolar PI(4)P signal is denser in the DFC than GC, but on the other hand, the nucleolar PI(4,5)P2 signal is denser in the fibrillar centre (FC) and DFC [18]. These data illustrate the possible functional differences between PI(4)P and PI(4,5)P2 in nucleolar processes. The phosphorylation and dephosphorylation of these two phospholipids might happen during rDNA transcription at the edge of FC/DFC and also during later stages of rRNA processing and ribosomal assembly. Additionally, we detected PI(4)P within the nuclear speckles (Figure 2a and Figure 3d). It localises inside of the nuclear speckles, avoiding dense regions marked by Son and it also localises at their edges (Figure 2a, inset). On the other hand, PI(4,5)P2 also localises to the nuclear speckles, but mostly inside the nuclear speckles rather than at their edges and it seems to localise more to the denser regions stained by Son (Figure 4c, [19]). Moreover, PI(4)P localisation during mitosis also differs as compared to PI(4,5)P2 localisation (Figure 6 and Figure S2). During mitosis, PI(4)P is dispersed, similarly to PI(4,5)P2, throughout the cytoplasm but does not localise to any larger structures or to NORs or MIGs, as PI(4,5)P2 does (Figure 6 and Figure S2, [18]). All our observed data support a functional difference between PI(4)P and PI(4,5)P2 in the cell nucleus. PI(4)P does not serve only as the precursor of PI(4,5)P2 but may also have a unique role in the cell nucleus. Nuclear PI(4)P might be generated by two possible pathways. PI4Kα and PI4Kβ kinases phosphorylate phosphatidylinositol (PI) to PI(4)P [47,48,49,50], and PTEN and SHIP phosphatases dephosphorylate PI(3,4)P2 and PI(4,5)P2 to PI(4)P [11,51,52,53,54,55,56]. These enzymes often localise to nuclear speckles [7,49,53,54,57] and nucleoli [50,58], which corresponds with the observed localisation of PI(4)P in nuclear speckles and nucleoli (Figure 1, Figure 2 and Figure 3). The majority of phosphoinositide-synthesising enzymes were shown to localise to the nuclear speckles (reviewed in [14]). Beside the enzymes, many phosphoinositides localise to nuclear speckles as well (PI(3)P, PI(4)P, PI(3,4)P2, PI(4,5)P2 and PI(3,4,5)P3) ([27] and our unpublished data). The function of the enzymes and the PIs in the nuclear speckles has not been determined yet. They may be involved in some nuclear processes taking place at the nuclear speckles, they may be stored there, or we speculate that nuclear speckles might be sites of nuclear phosphoinositide metabolism. As mentioned above, nuclear PI(4)P can be produced by two different pathways, so it is difficult to manipulate its levels in the nucleus. Using PI4K kinase inhibitors or down-regulating PI4K kinases by RNA interference decreases the PI(4)P level in the entire cell [26,59,60]. It would be problematic to interpret whether the phenotype is a result of a lowered PI(4)P level in the cytoplasm or that in the nucleus. The phenotype could also be a result of an increased level of other PIs as a response to the decreased level of PI(4)P. To selectively increase or decrease only the nuclear portion of PIs, it is possible to overexpress kinases or phosphatases with a nuclear localisation signal (NLS). We have tried to deplete nuclear PI(4,5)P2 by overexpressing phosphoinositide 5-phosphatase tagged with a NLS, and we were able to decrease the PI(4,5)P2 level by only 20% (unpublished data). It seems that the manipulation of nuclear PI metabolism results in the production of the downregulated PIs by the alternative pathway. For further studies, it will be challenging and crucial to find a reliable tool for the manipulation of the levels of PIs in vivo.

To come closer to elucidating the role of nuclear PI(4)P, we identified the possible nuclear protein interactors of PI(4)P using immunoprecipitation with the anti-PI(4)P antibody and the subsequent mass spectrometry analysis of nuclear extracts from asynchronous HeLa cells. We identified around 100 nuclear proteins playing a role in pre-mRNA processing, transcription, nuclear transport, rRNA processing ribosomal assembly and DNA replication and repair, which is in agreement with our observations of PI(4)P sub-localisation within the nucleus. Among the detected proteins in the experiment, 12 proteins were bound only to PI(4)P, in comparison with PI(4,5)P2 (RPA3, LMNB2, RBMS2, RRP15, TIA1, NACA, BTF3, ZMYND8, S100A7, TOR1AIP1, WBP4 and GNL3L) (Table S1, blue). These proteins are established as essential components of several nuclear processes, such as DNA replication, transcription and pre-mRNA splicing [61]. The rest of the identified proteins interacted with both PI(4)P and PI(4,5)P2, suggesting overlapping roles for both PIs. Indeed, PI(4)P is a precursor of PI(4,5)P2, or vice versa [14], indicating both PIs could have similar interaction partners. The phosphorylation and dephosphorylation of phosphoinositides might have a quick regulatory effect on their binding partners, resulting in different functional properties of the associated complexes. We confirmed and verified the interaction of the hnRNP U, NXF1 and NuMa proteins with PI(4)P and PI(4,5)P2 by two different tools, antibodies and PI-coated beads. We noted differences in the binding patterns of these proteins to PI(4)P and PI(4,5)P2, which could be a result of antibodies’ affinity to their respective epitopes and that of PI-coated beads to their baits. The interaction of NuMa with phosphoinositides was previously described [62]. HnRNP U plays a role in the initiation of RNA polymerase II transcription, and NXF1 is involved in the transport of mRNA from the cell nucleus [63,64]. These data indicate a potential role of PI(4)P and PI(4,5)P2 in RNA transcription and mRNA export through interaction with hnRNP U and NXF1.

We have previously identified a novel nuclear structure—nuclear lipid islets (NLIs) [19,65]. They are 40–100 nm nucleoplasmic structures rich in lipids with RNA, proteins and chromatin on the outside. The periphery of NLIs is associated with the RNA Pol II transcription machinery and probably serves as a structural platform facilitating the formation of Pol II transcription factories. Importantly, PI(4,5)P2 plays an essential role in NLIs, influencing the levels of active RNA Pol II-mediated transcription. To test whether PI(4)P is also a component of NLIs, we co-localised PI(4,5)P2, visualised by anti-PI(4,5)P2 antibody, and PI(4)P, visualised by the PH domain of the OSH1 protein [27]. Interestingly, we observed the distinct localisation of PI(4,5)P2 and PI(4)P in the nucleoplasm (Figure 4 and Figure 5c). The nucleoplasmic foci formed by PI(4)P are smaller than those formed by PI(4,5)P2 (Figure 5a–c), and we did not detect PI(4)P in NLIs (Figure 4 and Figure 5d). These data show that PI(4)P can be a part of different functional complexes than PI(4,5)P2.

In conclusion, we showed that PI(4)P localises to various nuclear compartments, including nuclear lamina, nucleoli, nuclear speckles and small nucleoplasmic foci. Moreover, we identified nuclear binding partners of PI(4)P, employing immunoprecipitation and mass spectrometry analysis. Based on the identified proteins, we suggest that PI(4)P participates in essential nuclear processes, such as replication, transcription and mRNA and rRNA processing. Further research is needed to determine the exact roles of PI(4)P in the cell nucleus, and this study could serve as a cornerstone for following studies revealing the functions of PI(4)P in the nucleus.

## Supplementary Materials

The following are available online at https://www.mdpi.com/2073-4409/9/5/1191/s1, Figure S1: Localisation of PI(4)P in the cell. Figure S2: PI(4)P and PI(4,5)P2 have a different localisation during mitosis, Table S1: List of the PI(4)P-binding partners.
