# Supplementary material for: Super-Resolution Localisation of Nuclear PI(4)P and Identification of Its Interacting Proteome
Source: Cells. 2020 May 11;9(5):1191. doi: 10.3390/cells9051191 (PMC7291030; doi:10.3390/cells9051191)
Supplement: Supplementary file 1 [file cells-09-01191-s001.zip › Supplement_2/Figures S1 and S2 legends.docx]

Figure S1: Localisation of PI(4)P in the cell. Nuclear and cytoplasmic focal planes of PI(4)P stained by anti-PI(4)P antibody and observed by confocal microscopy. DNA was stained by DAPI. Scale bars: 10 μm.

Figure S2: PI(4)P and PI(4,5)P2 have a different localisation during mitosis. Localisation of (a) PI(4)P with RPA194, a marker of the nucleoli (b) PI(4,5)P2 with RPA194, (c) PI(4,5)P2 and Son, a marker of the nuclear speckles, the localisation in telophase was observed by confocal microscopy. DNA was stained by DAPI. Scale bars: 10 μm.
