# Supplementary material for: Super-Resolution Localisation of Nuclear PI(4)P and Identification of Its Interacting Proteome
Source: Cells. 2020 May 11;9(5):1191. doi: 10.3390/cells9051191 (PMC7291030; doi:10.3390/cells9051191)
Supplement: Supplementary file 1 [file cells-09-01191-s001.zip › Supplement_2/Table S1_legend.docx]

Table S1: List of the PI(4)P-binding partners. There are three sheets: (i) all interactors – a complete list of PI(4)P interactors, nuclear proteins are highlighted in red (ii) nuclear interactors - a list of nuclear interactors derived from the list of all interactors, with enrichment log2(fold change) > 0.5 and a number of unique peptides identified ≥ 2. Proteins found only in the PI(4)P fraction are highlighted in blue, proteins enriched more in PI(4)P fraction than PI(4,5)P2 fraction are highlighted in orange (iii) legend – a description of results.
