# Supplementary figures and images for: Super-Resolution Localisation of Nuclear PI(4)P and Identification of Its Interacting Proteome
Source: Cells. 2020 May 11;9(5):1191. doi: 10.3390/cells9051191 (PMC7291030; doi:10.3390/cells9051191)

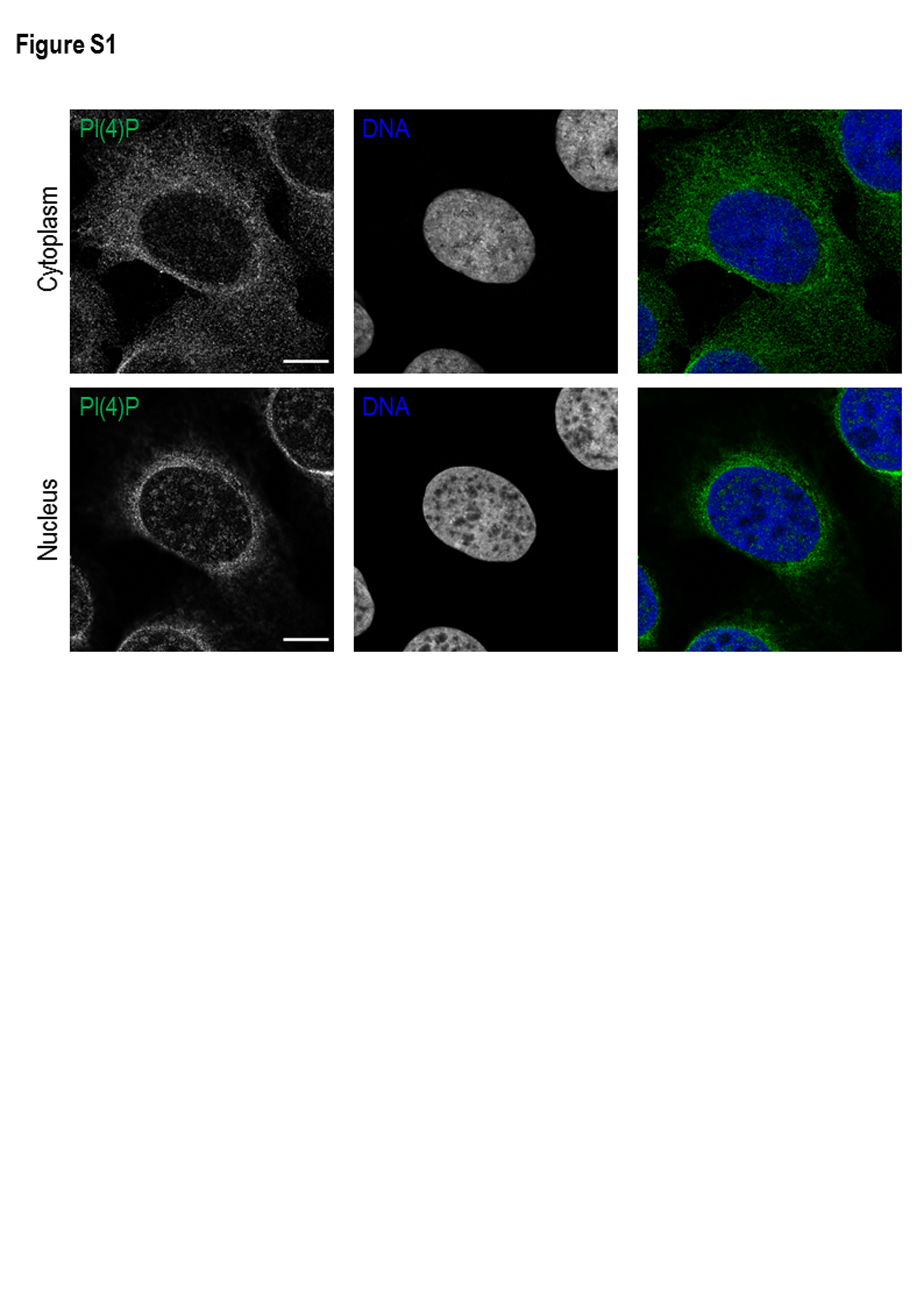

Supplement: Supplementary file 1 [file cells-09-01191-s001.zip › Supplement_2/Figure S1.tif]

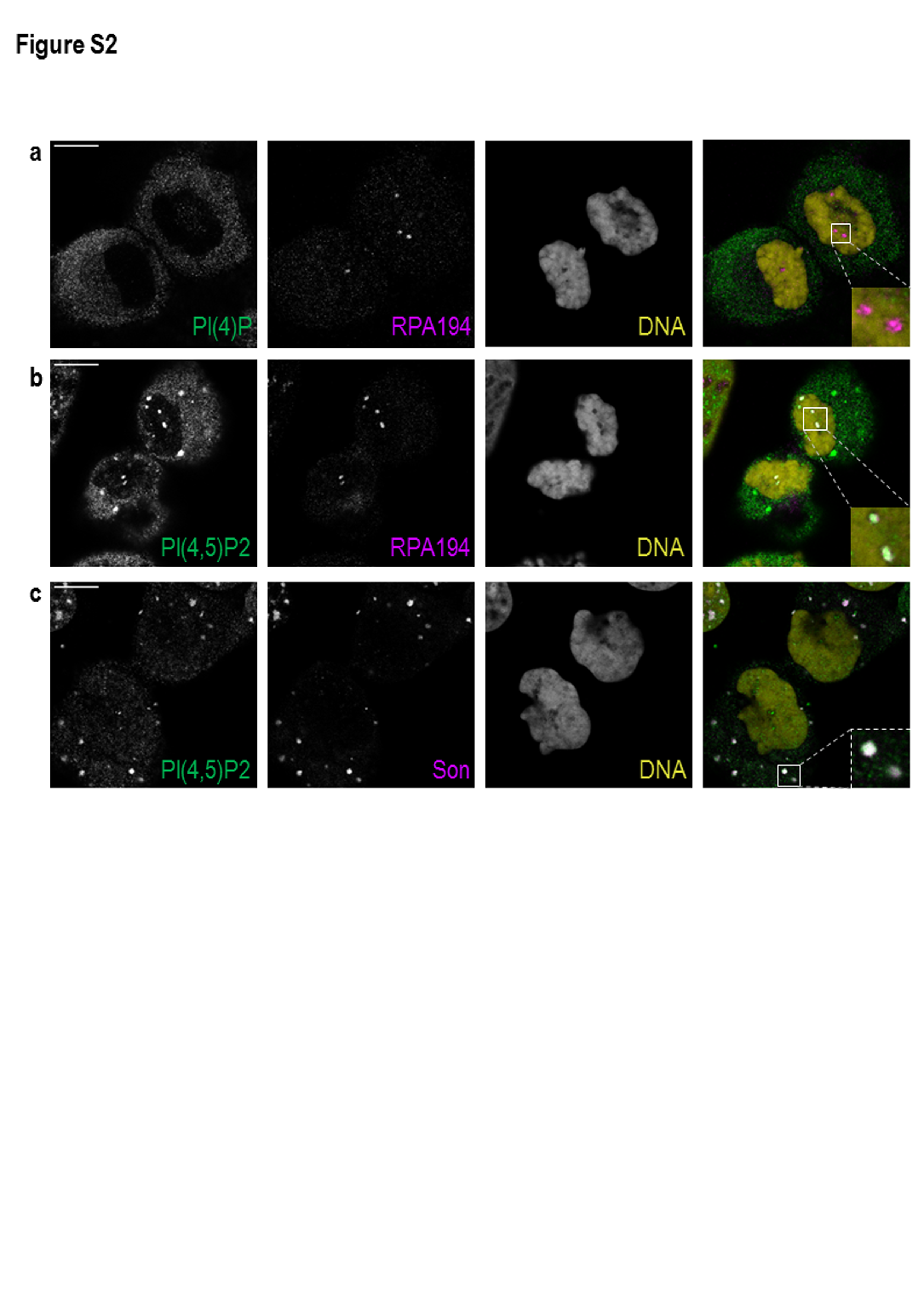

Supplement: Supplementary file 1 [file cells-09-01191-s001.zip › Supplement_2/Figure S2.tif]
